# Energy metabolism and mitochondrial defects in X-linked Charcot-Marie-Tooth (CMTX6) iPSC-derived motor neurons with the p.R158H PDK3 mutation

**DOI:** 10.1038/s41598-020-66266-5

**Published:** 2020-06-05

**Authors:** G. Perez-Siles, A. Cutrupi, M. Ellis, R. Screnci, D. Mao, M. Uesugi, Eppie M. Yiu, Monique M. Ryan, B. O. Choi, G. Nicholson, M. L. Kennerson

**Affiliations:** 1 Northcott Neuroscience Laboratory, ANZAC Research Institute, Sydney, Australia; 2 Sydney Medical School, University of Sydney, Sydney, Australia; 3 Molecular Medicine Laboratory, Concord Repatriation General Hospital, Sydney, Australia; 4 School of Life Sciences, University of Technology Sydney, Sydney, NSW Australia; 5 Institute for Integrated Cell-Material Sciences and Institute for Chemical Research, Kyoto University, Kyoto, Japan; 6 Department of Neurology, Royal Children’s Hospital, Flemington Road, Parkville, VIC Australia; 7 Neuroscience Research, Murdoch Children’s Research Institute, Melbourne, VIC Australia; 8 Department of Paediatrics, The University of Melbourne, Melbourne, VIC Australia; 9 Department of Neurology, Samsung Medical Center, Sungkyunkwan University School of Medicine, Seoul, Korea

**Keywords:** Cell biology, Molecular biology, Neuroscience, Stem cells, Diseases, Medical research, Pathogenesis

## Abstract

Charcot-Marie-Tooth (CMT) is a group of inherited diseases clinically and genetically heterogenous, characterised by length dependent degeneration of axons of the peripheral nervous system. A missense mutation (p.R158H) in the pyruvate dehydrogenase kinase 3 gene (*PDK3*) has been identified as the genetic cause for an X-linked form of CMT (CMTX6) in two unrelated families. PDK3 is one of four PDK isoenzymes that regulate the activity of the pyruvate dehydrogenase complex (PDC). The balance between kinases (PDKs) and phosphatases (PDPs) determines the extend of oxidative decarboxylation of pyruvate to generate acetyl CoA, critically linking glycolysis and the energy producing Krebs cycle. We had shown the p.R158H mutation causes hyperactivity of PDK3 and CMTX6 fibroblasts show hyperphosphorylation of PDC, leading to reduced PDC activity and ATP production. In this manuscript we have generated induced pluripotent stem cells (iPSCs) by re-programming CMTX6 fibroblasts (iPSC^CMTX6^). We also have engineered an isogenic control (iPSC^isogenic^) and demonstrated that genetic correction of the p.R158H mutation reverses the CMTX6 phenotype. Patient-derived motor neurons (MN^CMTX6^) show increased phosphorylation of the PDC, energy metabolism defects and mitochondrial abnormalities, including reduced velocity of trafficking mitochondria in the affected axons. Treatment of the MN^CMTX6^ with a PDK inhibitor reverses PDC hyperphosphorylation and the associated functional deficits founds in the patient motor neurons, demonstrating that the MN^CMTX6^ and MN^isogenic^ motor neurons provide an excellent neuronal system for compound screening approaches to identify drugs for the treatment of CMTX6.

## Introduction

Charcot-Marie-Tooth neuropathy (CMT) is a genetically heterogeneous group of inherited diseases characterised by length dependent axonal degeneration of the motor and sensory neurons of the peripheral nervous system. CMT is the most common inherited neurological disorder with a prevalence of 1 in 2500 people^1^. Genetically, more than 1000 mutations in over 80 genes have been found to date^2^. Our laboratory identified a missense mutation (c.G473A p.R158H) in the pyruvate dehydrogenase kinase 3 (*PDK3*) gene causing an X-linked form of CMT, designated as CMTX6^3^. The same genetic mutation has been found in a second unrelated family, suggesting the specific nucleotide transition may be a hotspot for the R158H mutation^4^.

PDK3 negatively regulates the pyruvate dehydrogenase complex (PDC) activity in the mitochondria by reversible phosphorylation of its first catalytic component (E1). PDK3 hence has a fundamental role in linking glycolysis to the energy producing Krebs cycle. The discovery of *PDK3* has added to the growing list of CMT genes related to mitochondrial biology^5^, suggesting mitochondrial pathway deficits may be a common theme in some CMT neuropathies. Our previous investigations showed the p.R158H mutation produces a PDK3 enzyme with increased kinase activity^3^. Experiments using CMTX6 patient fibroblasts demonstrated mutant PDK3 hyperactivity leads to increased phosphorylation of the PDC E1 subunit at specific serine residues and hence attenuation of the pyruvate dehydrogenase activity. Consequently, CMTX6 patient fibroblasts show increased lactate, decreased ATP and alteration of the mitochondrial network. Importantly, E1 hyperphosphorylation was reversed by treating the patient fibroblasts with a pan PDK inhibitor, dichloroacetic acid (DCA), opening a venue for therapeutic intervention for CMTX6^6^.

Despite the active research for therapies that can stop or ameliorate degeneration of axons, there is yet no cure for CMT. This fact can be explained in part by a reliance on animal models, transformed cell lines and heterologous recombinant systems for drug discovery^7^. The use of human induced pluripotent stem cell (iPSC) technology has recently opened up the possibility to produce disease-relevant human models for drug discovery for inherited diseases in general and neurodegenerative disorders in particular^8^. iPSC lines from CMT patients have increasingly been generated^9–11^ and key pathological features for the disease have been replicated in some instances^12–15^ in CMT patient derived motor neurons.

In this study we have used patient fibroblasts from a recently identified family carrying the p.R158H PDK3 mutation^4^ and, following confirmation of the E1 hyperphosphorylation as a CMTX6 disease signature, generated iPSCs from this patient. To eliminate the influence of variable genetic backgrounds from genetically unrelated controls, we also generated an isogenic wild type iPSC line by targeted gene correction using the CRISPR/Cas9 system. Our results show the E1 hyperphosphorylation is maintained in the CMTX6-derived iPSCs following reprograming of the patient fibroblasts and is also observed after differentiation into spinal cord motor neurons. Our data reveals abnormalities in the bioenergetic profile and mitochondrial morphological features in the CMTX6-derived motor neurons. Additionally, analyses of the organelle trafficking demonstrated the PDK3 mutation specifically affects mitochondrial trafficking in the patient motor neurons. Importantly, we have reversed the CMTX6 cellular phenotype both pharmacologically, using a pan PDK inhibitor, and by genetically correcting the p.R158H PDK3 mutation.

## Material and Methods

### Research guidelines and regulations

All research and cell culture procedures were conducted following written consent according to protocols approved by the Sydney Local Health District Human Ethics Review Committee, Concord Repatriation General Hospital, Sydney, Australia (reference number: HREC/11/CRGH/105). Informed consent for study participation was obtained from all patients and controls. All research was performed in accordance with relevant guidelines and regulations.

### Fibroblasts culture

Primary fibroblasts were cultured from patient skin biopsies and maintained at 37 °C in humidified air and 5% CO_2_ as per standard practice^16^. Fibroblast cell culture medium: DMEM (Gibco, Life technologies) supplemented with 10% (v/v) fetal bovine serum (SAFC Biosciences), 1% (v/v) Penicillin Streptomycin (Gibco, Life technologies) and 1% (v/v) L-glutamine (Gibco, Life technologies).

### Patient iPSC generation

Reprogramming was performed by FUJIFILM Cellular Dynamics as previously described^17^. Briefly, fibroblasts obtained from a CMTX6 patient harbouring the p.R158H mutation in the *PDK3* gene were transfected using oriP/EBNA-1-based vectors^18^ using the Lonza VPD-1001 Human Dermal Fibroblast Nucleofector Kit and then placed on matrigel-coated plates in reprogramming medium^17^ for 1 week followed by Essential 8^TM^ Medium (E8) for an additional 2 weeks. The iPSC colonies were singly picked and propagated with E8 on matrigel-coated plates. The iPSCs were confirmed to be karyotypically normal by G-banded karyotyping (WiCell). The pluripotency of the iPSC lines was confirmed by their expression of endogenous pluripotent stem cell genes and the identity of the iPSCs was matched to the starting fibroblast line (FUJIFILM Cellular Dynamics).

### Targeted gene correction in iPSC^R158H^ by CRISPR/Cas9

The iPSC^CMTX6^ patient line was corrected using nuclease-mediated engineering (FUJIFILM Cellular Dynamics, Inc.). A nuclease was designed to target the genome in the *PDK3* gene, and an oligonucleotide donor DNA molecule centered on the modification site was used as a template for the correction. An additional single silent base change (c.C471T) was introduced to prevent nuclease re-cutting during the CRISPR/Cas9 mediated gene correction. Plasmid DNA encoding the nuclease was electroporated along with a 60 nucleotide single stranded oligonucleotide donor molecule (IDT, Coralville IA) into the iPSC line. After recovery and outgrowth single cell sorting was performed and allele specific PCR was used to identify targeted clones. Clones were expanded for confirmation of correct gene editing at amino acid 158. The initial patient sequence at positions 157 and 158 of AAC-CAC (N157-H158) was converted to AAT-CGG (N157-R158) and contained silent mutations to aid in screening.

### Motor neuron differentiation

The motor neuron differentiation protocol is based on dual SMAD inhibition and was performed as previously described^12^ with modifications introduced by our laboratory to increase efficacy of the procedure. Briefly, colonies were expanded as adherent cultures on matrigel-coated culture plates to 90 to 95% confluence. Cells were then switched to knockout serum replacement medium containing 1 μM Dorsomorphin dihydrochloride (STEMCELL) and 10 μM SB431542 (STEMCELL) and after 3 days, gradually changed to N2 medium. On day 7, cultures were changed to a motor neuron maturation medium consisting of DMEM/F12 supplemented with B27, N2, 0.8 mM ascorbic acid, 1.5 μM retinoic acid, 200 nM Smoothened Agonist (EMD Chemicals), 2 ng/mL BDNF, 2 ng/mL CTNF and 2 ng/mL GDNF (Life Technologies) and matured for 10 days. Cultures were then dissociated using 1 U/ml Dispase (STEMCELL) and terminal differentiation achieved by a three-dimensional (3D) culture system using ultra-low attachment in 6 well plates (Corning-Costar) for an additional 12 days with replacement of the maturation media every second day. Maturation of motor neurons in these conditions facilitated aggregation of cells in homogeneous spheres that were subsequently dissociated into single cells using Accutase and subsequently seeded on matrigel coated plates for final maturation in the presence of 0.01 μM SN38-P for decontaminating the culture of proliferative stem cells^19^.

### Immunofluorescence

Immunodetection was performed following standard procedures as previously described^20^. Briefly, cells were washed once using PBS, fixed with 4% paraformaldehyde for 12 min at room temperature (RT), permeabilized in phosphate-buffered saline (PBS) containing 0.3% (v/v) Triton X-100 and blocked in 5% (w/v) bovine serum albumin (BSA) for 60 min. Cells were incubated with primary antibodies overnight in 4 °C. The antibodies used in immunofluorescence assay are as follows: anti-SOX2 (Cell Signalling, #3579, 1:400), anti-NANOG (Cell Signalling, #4903, 1:400), anti-OCT4 (Cell Signalling, #2840, 1:400), anti-TUJ1 (Sigma, T2220, 1:500), anti-NF68 (Abcam, 8186, 1:1000), anti-HB9 (Millipore, ABN174, 1:500), (anti-ISL1 (DSHB, ab20670 1:150), anti-PAX6 (DSHB, 81.5C10, 1:200), anti-pSer293 (Millipore, AP1062, 1:250), antipSer300 (Millipore, ABS194, 1:250). Alexa Fluor secondary antibodies (Invitrogen) (Alexa Fluor were used at 1:500 dilution and incubated for 2 h at RT. Nuclei were stained with 300 nM 4,6-diamidino-2- phenylindole (DAPI, Molecular Probes) and mounted using Prolong Gold antifade reagent (Invitrogen). When required, cells were incubated with 200 nM MitoTracker Red CMX Red (Invitrogen) to visualize mitochondrial structures and treated with the PDK inhibitor dichloroacetic acid DCA (Sigma-Aldrich) at 5 mM for 1 h before staining. Cells were visualized using a Leica SP8 confocal microscope and images acquired at 63X magnification.

### Quantification of E1 phosphorylation within mitochondria

For quantification of the E1 phosphorylation levels at the specific Ser sites, iPSCs and MNs were incubated with 100 nM MitoTracker Deep Red FM (Invitrogen) for 1 h prior to staining with anti-pSer293 or anti-pSer300 antibodies as described in section 2.6 above. To precisely define the region for each image corresponding to the mitochondria, a region of interest (ROI) was defined using the image processing package *Fiji*^21^. Briefly, a threshold was applied to the channel containing the mitochondria information to set appropriate signal-to-noise ratios. By using the “Analyze Particles” option, the region corresponding to the mitochondria was outlined in every channel (size: 0.1 – Infinity; circularity (0.0–0.5). Using the multi measure option of the ROI master tool, the intensity of the signal corresponding to PSer293 or PSer300 within mitochondria was calculated. 10 images were taken for each condition at 63X magnification using a Leica SP8 confocal microscope.

### RT-qPCR

RNA was extracted from confluent iPSC colonies using the RNeasy mini kit (Qiagen). RNA (1 μg) was reverse transcribed using the High-Capacity cDNA Reverse Transcription Kit (Applied Biosystems) following the manufacture’s instructions. Quantitative RT-PCR was performed with TaqMan Gene Expression Assays using the following probes: CDH1 (Hs01023895_m1), LIN28 (Hs00702808_s1), FOXD3 (Hs00255287_s1), NANOG (Hs02387400_g1), TDGF1 (Hs02339499_g1) and GAPDH (Hs02786624_g1). To qualitatively assess the differential expression of the pluripotency genes in the iPSC lines versus the CMTX6 patient fibroblasts, raw Ct values are presented^22^.

### Western blotting

Immunoblotting was performed following standard procedures^23^. Briefly, cell lysates were obtained from iPSC lines growing at confluency using RIPA buffer (50 mM Tris-HCl pH 8.0, 150 mM NaCl, 0.1% w/v SDS, 1% v/v Triton X-100, 1% w/v Sodium deoxycholate, 1X cOmplete, Mini EDTA-free protease inhibitor). After protein determination (Pierce BCA Protein Assay Kit, ThermoScientific) cell lysates (15 μg) were subjected to SDS-polyacrylamide gel electrophoresis and transferred to polyvinylidene difluoride (PVDF) membranes. Membranes were probed with anti-SOX2 (Cell Signalling, #3579, 1:400), anti-NANOG (Cell Signalling, #4903, 1:400), anti-OCT4 (Cell Signalling, #2840, 1:400) antibodies. β-actin (Cell Signaling #4967 S) was used as loading control. Anti-rabbit (SIGMA Aldrich) and anti-mouse (Abcam) horseradish peroxidase (HRP) conjugated secondary antibodies were used and signal detected by adding a chemiluminescent substrate (Merck).

### Bioenergetic assessment of iPSC-derived motor neurons

Mitochondrial oxygen consumption rates (OCR) in differentiated motor neurons were measured using a XF24 Seahorse Biosciences Extracellular Flux Analyzer as previously described^24^. Following differentiation of the patient and isogenic motor neurons, 60,000 cells per well were plated onto polyornithine- and laminin-coated Seahorse 24-well plate, grown for additional 7 days and continued maturation with the above indicated media. 45 min prior to initiating the assay, maturation media was replaced with 500 μl of XF unbuffered media supplemented with 10 mM glucose, 1 mM pyruvate and 2mM L-Glutamine and the cells were incubated at 37 °C to allow media temperature and pH to reach equilibrium. Seahorse analyzer injection ports contained (A) 1 μM oligomycin A, an ATP synthase inhibitor to inhibit OXPHOS and test respiration coupling to ATP synthesis; (B) 1 μM FCCP, a protonophore uncoupling agent to increase respiration rate; (C) 1 μM rotenone/ antimycin, inhibitors of mitochondrial respiratory complex I and complex III respectively to cause complete inhibition of mitochondrial respiration and hence determine maximal mitochondrial OCR.

### ATP assay

50,000 cells per well were plated onto a matrigel-coated 96-well plate, grown for an additional 7 days for continued maturation. Cellular ATP was measured using an ATPlite assay kit (PerkinElmer, Massachusetts, UK). Briefly, maturation media was removed from wells and mammalian cell lysis solution (50 μl) was added and the plate incubated for 10 min with agitation at 37 °C. Substrate solution (50 μl) was added to the cell lysate and incubated in the absence of light for 10 min. Luminescence was measured on an EnSpireMultimode Plate Reader (PerkinElmer) and data was represented as arbitrary luminescence units (ALU) for each line.

### Live cell imaging

Following dissociation of neurospheres with Accutase, 50,000 motor neurons were plated onto polyornithine- and laminin-coated CellCarrier-96 Ultra Microplates (PerkinElmer). At day 32 of differentiation, the cells were stained with 200 nM MitoTracker Deep Red (Invitrogen) or 100 nM LysoTracker Deep Red (Invitrogen) for 45 min and then replaced with fresh medium. Live-cell imaging was performed using a Leica SP8 confocal microscope equipped with an incubation chamber to maintain the cells at 37 °C and 5% C0_2_ at all times. Images were obtained at 63X magnification with 2-s intervals for 5-min, yielding 150 frames. Kymographs were built using the KymographBuilder plugin in *Fiji*. Trafficking parameters of all particles (track displacement and velocity) were subsequently obtained using the web-based software KymoButler^25^ version 1.1.1 that can be accessed in the following link: https://www.wolframcloud.com/objects/deepmirror/Projects/KymoButler/KymoButlerForm Only particles successfully tracked for at least 60 s were considered for all subsequent calculations shown in the *Results* sections.

## **Results**

### Confirmation of E1 hyperphosphorylation phenotype in the CMTX6 patient fibroblasts

We previously showed the p.R158H substitution confers mutant PDK3 enzyme hyperactivity and stronger binding affinity than the wild type PDK3 for the inner-lipoyl (L2) domain in the E2 chain of the PDC^3^. Our follow up studies demonstrated skin fibroblasts cultured from the original Australian CMTX6 family show increased phosphorylation of the E1 subunit at Ser^293^ and Ser^300^ sites, causing downregulation of dehydrogenase activity and subsequent accumulation of lactate, reduced production of cellular ATP and mitochondria abnormalities. Our recent discovery of a second unrelated family harbouring the same genetic mutation in the *PDK3* gene, provided the opportunity to confirm these pathological consequences and clearly define a CMTX6 specific cellular signature in the patient cells.

**Figure 1:**
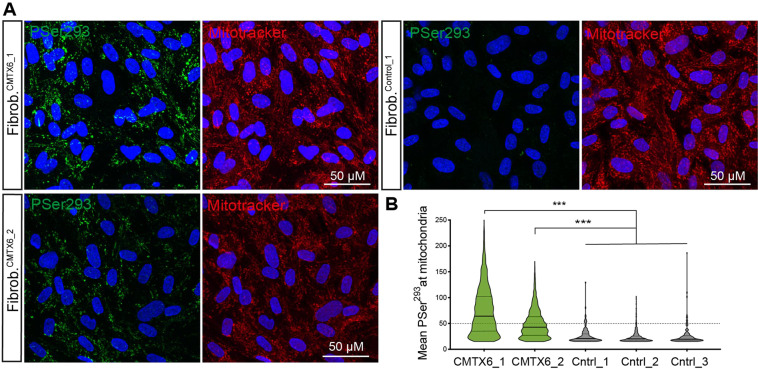
E1 hyperphosphorylation is a CMTX6 disease phenotype. (**A**) CMTX6 fibroblasts from 2 independent families (Fibr^CMTX6_1^ and Fibr^CMTX6_2^) show increased levels of E1 phosphorylation at Ser^293^ (green) within the mitochondria (red) when compared to control fibroblasts Fibr^Ctrl_1^. (**B**) Quantification of the mean fluorescence of the PSer^293^ staining within the region of interest (ROI) defined by staining the cells with MitoTracker Red. Violin plot shows full distribution of all data points acquired (n > 5000 mitochondria) for the 2 patient-derived fibroblast lines and 3 neurologically normal lines (Fibr^Ctrl_1,2,3^). *p* values were obtained by ANOVA followed by Tukey’s post hoc test (*** *p* < 0.0005).

Skin fibroblasts from a family member of the newly identified CMTX6 Korean family (Fibr^CMTX6_1^) and 3 lines of neurologically normal individuals (Fibr^Ctrl1,2,3^) were cultured and phosphorylation levels of the E1 subunit serine residues Ser^293^ assessed by immunofluorescence (Fig. 1A). Fibroblasts from the original Australian CMTX6 family were used as a positive control (Fibr^CMTX6_2^). This experiment showed Ser^293^ had increased phosphorylation in the CMTX6 derived fibroblasts when compared to control cells. Quantification of the images confirmed the confocal microscopy observations, with statistically significant increases of E1 phosphorylation in the two unrelated Fibr^CMTX6^ lines at the Ser^293^ when compared with Fibr^Ctrl^ lines (Fig. 1B). Phosphorylation of the E1 subunit is therefore a pathological signature of the CMTX6 p.R158H mutation.

### Establishment of a patient iPSC^R158H^ line and targeted gene correction of the PDK3 mutation with CRISPR/Cas9 system

**Figure 2:**
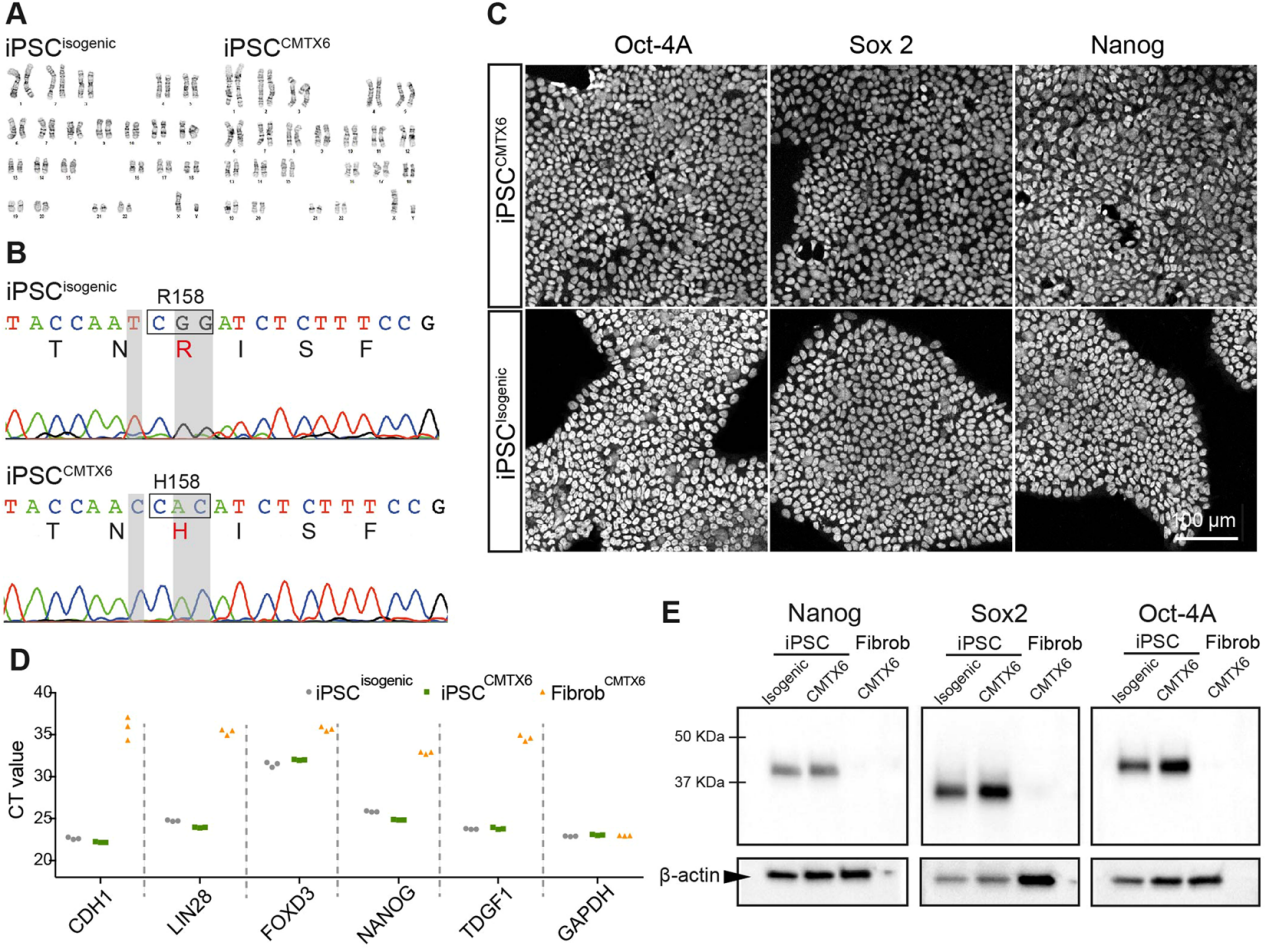
CMTX6 patient-derived iPSCs harbouring the p.R158 PDK3 mutation, have a normal karyotype and display cellular features of pluripotency. (**A**) Karyotyping and g-band analysis show the iPSC clones have a normal 46,XY karyotype. (**B**) DNA sequencing confirmed the c.G473A nucleotide change in the patient iPSC^CMTX6^ line. Grey shadow highlights the corrected bases in the engineered line. The 2 nucleotide changes within the mutated codon restored the R158 residue in the iPSC^Isogenic^ line. An additional silent change c.C471T adjacent to the mutated amino acid residue was introduced to prevent nuclease re-cutting during the CRISPR/Cas9 mediated gene correction. (**C**) Immunofluorescent staining confirms expression of Oct-4A, Sox2 and Nanog in the two iPSC lines. (**D**) Real-Time PCR shows increased differential expression of *CDH1, LIN28, NANOG* and *TDGF1* genes in iPSC^Isogenic^ (grey) and iPSC^CMTX6^ (GREEN) lines relative to skin fibroblasts (orange). (**E**) Western blot demonstrates expression of pluripotency markers in the iPSCs and not in the CMTX6 patient fibroblasts. Boxed images have been obtained from the same blot and assembled for this figure.

Skin fibroblasts from the CMTX6 Korean patient were reprogrammed using non-integrative episomal plasmids by FUJIFILM Cellular Dynamics International (CDI) using company *in house* protocols. The CMTX6 causing mutation c.G473A was repaired in the iPSC^CMTX6^ line using the CRISPR/Cas9 system by CDI to generate an isogenic control (iPSC^Isogenic^) that preserves the genetic background of the CMTX6 patient. Karyotyping and G-banding analysis showed that both, patient iPSC^R158H^ and genetically corrected iPSC^Isogenic^, maintained a normal 46,XY karyotype (Fig. 2A). The presence of the CMTX6 disease-causing mutation and correction of the R158H amino acid change was confirmed by genomic DNA sequencing (Fig. 2B). An additional single silent base change (c.C471T) in the codon corresponding to amino acid 157 was introduced in the isogenic line to prevent nuclease re-cutting during the CRISPR/Cas9 mediated gene correction. Pluripotent characteristics of the iPSC lines were confirmed by three experimental approaches: iPSC colonies stained positive for the pluripotency markers OCT4A, SOX2 and NANOG (Fig. 2C) and protein expression of these transcription factors was absent (Fig. 2D) in the originating fibroblast lines (Fibr^CMTX6^). Quantitative RT-PCR revealed that endogenous pluripotency-associated genes, including *CDH1, LIN28, FOXD3, NANOG* and *TDGF1*, were expressed at higher levels in the iPSCs when compared to the Fibr^CMTX6^ cells (Fig. 2E).

### Patient iPSC^CMTX6^ line displays pathogenic features associated with CMTX6 patient fibroblasts and is corrected after targeted gene correction in the iPSC^isogenic^ line

**Figure 3:**
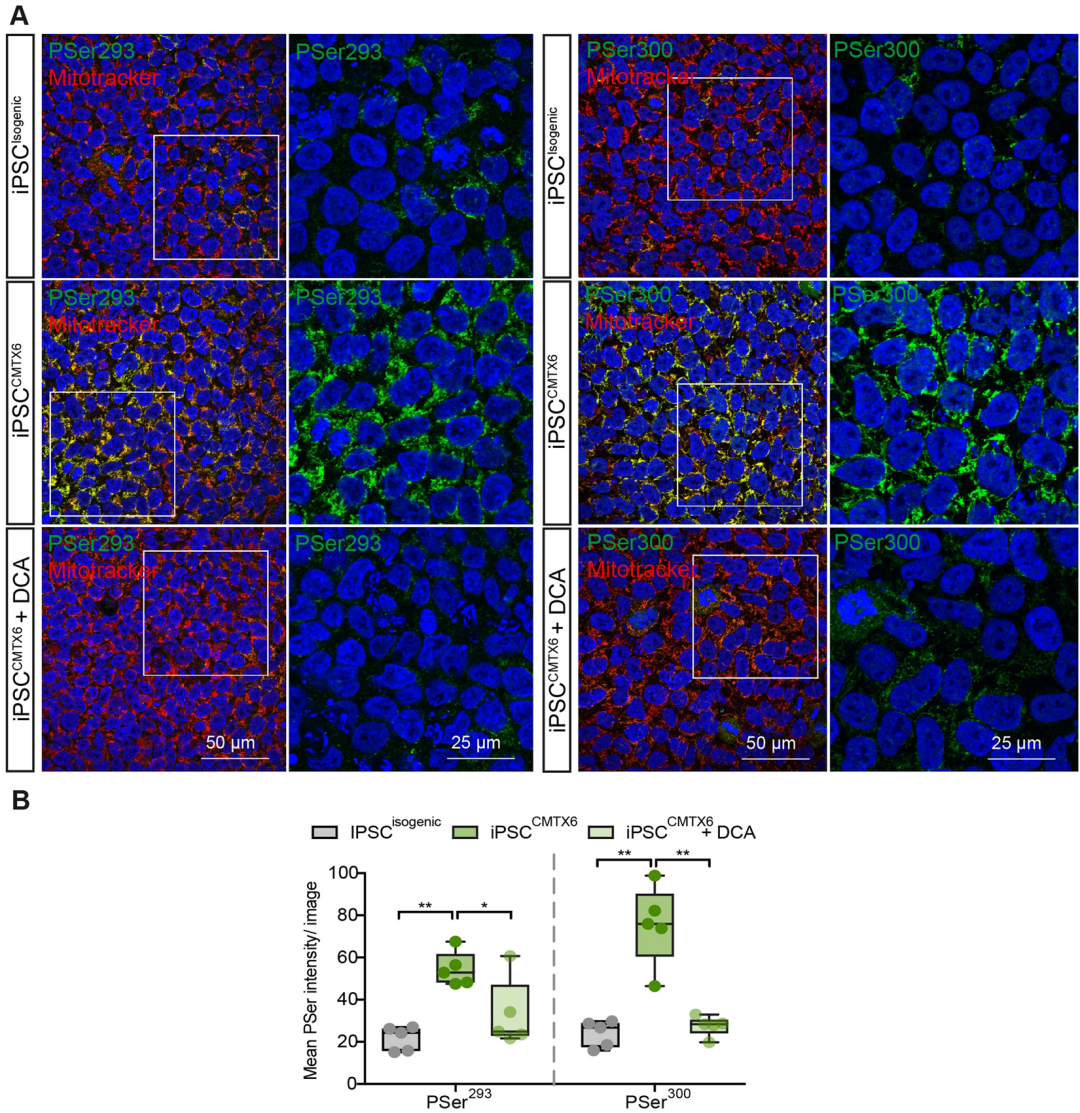
Molecular phenotype of the iPSC lines. (**A**) Cells stained with E1-PSer293 and E1-PSer300 antibodies (green), Mitotracker Red and DAPI (blue). Boxed area is shown enlarged in the right column. Where indicated, iPSC^CMTX6^ line was treated with DCA 5 mM for 1 h prior to staining; (**B**) Mean fluorescence within the region of interest (defined by staining the cells with MitoTracker) was calculated for PSer293 and PSer300 on iPSC^isogenic^ (grey), iPSC^CMTX6^ (green) and iPSC^CMTX6^ following DCA treatment (light green). Each data point represents the averaged mean fluorescence calculated in individual images containing at least 100 cells. *p* values were obtained by ANOVA followed by Tukey’s post hoc test (*p < 0.05; **p < 0.005).

Increased phosphorylation of the E1 subunit at Ser^293^ and Ser^300^ represents a CMTX6 phenotypic signature (Fig. 1A). Determining whether E1 hyperphosphorylation is maintained in the iPSC^CMTX6^ after reprograming of the original Fibr^CMTX6^ is a fundamental question for the purpose of disease modelling. Phosphorylation of E1 is a reversible process. Physiologically, pyruvate dehydrogenase phosphatases (PDP) remove phosphate groups from Ser sites hence rendering the PDC complex active. E1 phosphorylation can also be reverted pharmacologically and our previous investigations had shown this could be achieved by treating the CMTX6 patient fibroblasts with the pan PDK inhibitor dichloroacetate (DCA). To assess levels of E1 phosphorylation in the iPSC^CMTX6^ and iPSC^Isogenic^ lines and evaluate the potential for pharmacologically reverting the CMTX6 phenotype, iPSC colonies were stained with PSer^293^ and PSer^300^ specific antibodies. The iPSC^CMTX6^ line was also treated with DCA prior to immunofluorescence analysis. Figure 3 demonstrates the CMTX6 hyperphosphorylation phenotype is observed in the iPSC^CMTX6^ line (middle row) at Ser^293^ (left panel) and Ser^300^ (right panel). Importantly, gene editing of the p.R158H mutation leads to a reduction in the E1 phosphorylation in the iPSC^Isogenic^ line at the two Ser sites (upper row). Moreover, treatment of the patient iPSC^CMTX6^ cells with DCA (bottom row) also reduced phosphorylation of E1 subunit to levels found in the iPSC^Isogenic^ line. Quantification of the intensity of the phosphorylated Ser residues within a region defined by staining of the cells with MitoTracker, confirmed the confocal microscopy observations reached statistical significance (Fig. 3B).

### Differentiation of CMTX6-derived and isogenic iPSC lines into spinal cord motor neurons

**Figure 4:**
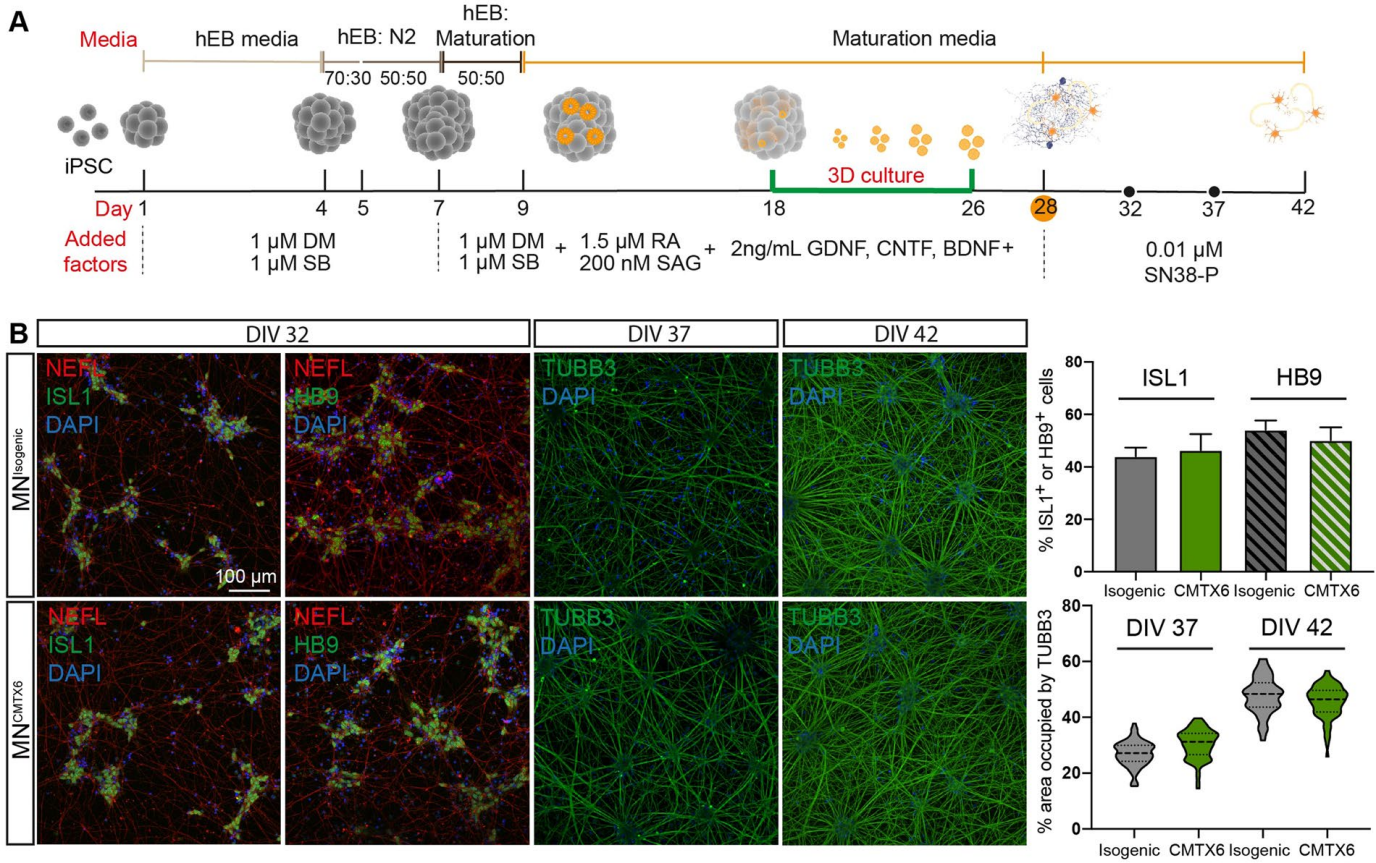
Differentiation of CMTX6 patient-derived and control iPSCs into motor neurons. (**A**) Timeline for spinal cord motor neuron differentiation from iPSC following a dual SMAD inhibition protocol. Diagram shows media used and factors added throughout the process. Cells were grown in suspension (3D) between days 18 and 26. Abbreviations: DM (Dorsomorphin); SB (SB431542); RA (Retinoic acid); SAG (Smoothened agonist). GDNF (Glial cell line-derived neurotrophic factor); CNTF (Human Ciliary neurotrophic factor); BDNF (Brain-derived neurotrophic factor); phosphorylated 7-ethyl-10-hydroxycamptothecin (SN38-P). (**B**) Differentiated motor neurons show a robust expression of ISL1, HB9 and NEFL markers at DIV 32. Motor neurons organise into clusters of cell bodies at longer maturation times (DIV 37, DIV 42) with connecting axons (TUBB3) occupying larger areas of the culture.

Axonal degeneration of motor neurons from the peripheral nervous system is the pathological hallmark of CMT. To model CMTX6 *in vitro*, iPSC^CMTX6^ and iPSC^Isogenic^ lines were differentiated into spinal cord motor neurons based on protocols previously established by Saporta *et al*.^12^ to generate neuronal models of axonal CMT. This protocol is achieved by dual SMAD signalling inhibition (Dorsomorphin and SB431542) and neuronal patterning triggered with retinoic acid and activation of the Sonic Hedgehog pathway with Smoothened Agonist (SAG). Addition of glial cell line-derived neurotrophic factor (GDNF), ciliary neurotrophic factor (CNTF) and brain-derived neurotrophic factor (BDNF) collectively contribute to mature motor neuron development (Fig. 4A).

In our protocol, adapted from Daisuke Shimojo and colleagues^26^, maturation of differentiating motor neurons takes place in a three-dimensional (3D) culture system between days 18 and 26 of differentiation *in vitro* (DIV). In these conditions, differentiating motor neurons aggregate as suspended spheres (neurospheres). On 26 DIV neurospheres are dissociated into single cells and cultured in the presence of 0.01 μM SN38-P from 28 DIV onwards for the removal of proliferative stem cells and neuroprogenitors^19^. These 2 consecutive steps significantly upregulate the induction of somatic motor neurons in the differentiation process (Perez-Siles *et al*. 2019), demonstrated by the efficient induction of HB9/ISL-1, markers for somatic motor neurons, and the expression of the neuronal cytoskeletal proteins NEFL/βIII-Tubulin (Fig. 4B). CMTX6-derived motor neurons (MN^CMTX6^) showed similar neurite structures when compared to control motor neurons (MN^Isogenic^) at DIV 32, indicating that the differentiation potential of the CMTX6-derived cells is not affected by the PDK3 mutation. Maturation of MN^CMTX6^ and MN^Isogenic^ for an additional 5 days (37 DIV) and 10 days (42 DIV) resulted in the motor neuron cell bodies organizing into clusters, with individual axons establishing neuronal bundles. In these conditions, we found no differences in the ability of control and patient motor neurons to establish neuronal networks (Fig. 4B). In addition, no changes in axonal integrity or signs of degeneration were observed at these time-points.

### CMTX6-derived motor neurons show E1 hyperphosphorylation that can be pharmacologically reverted

**Figure 5:**
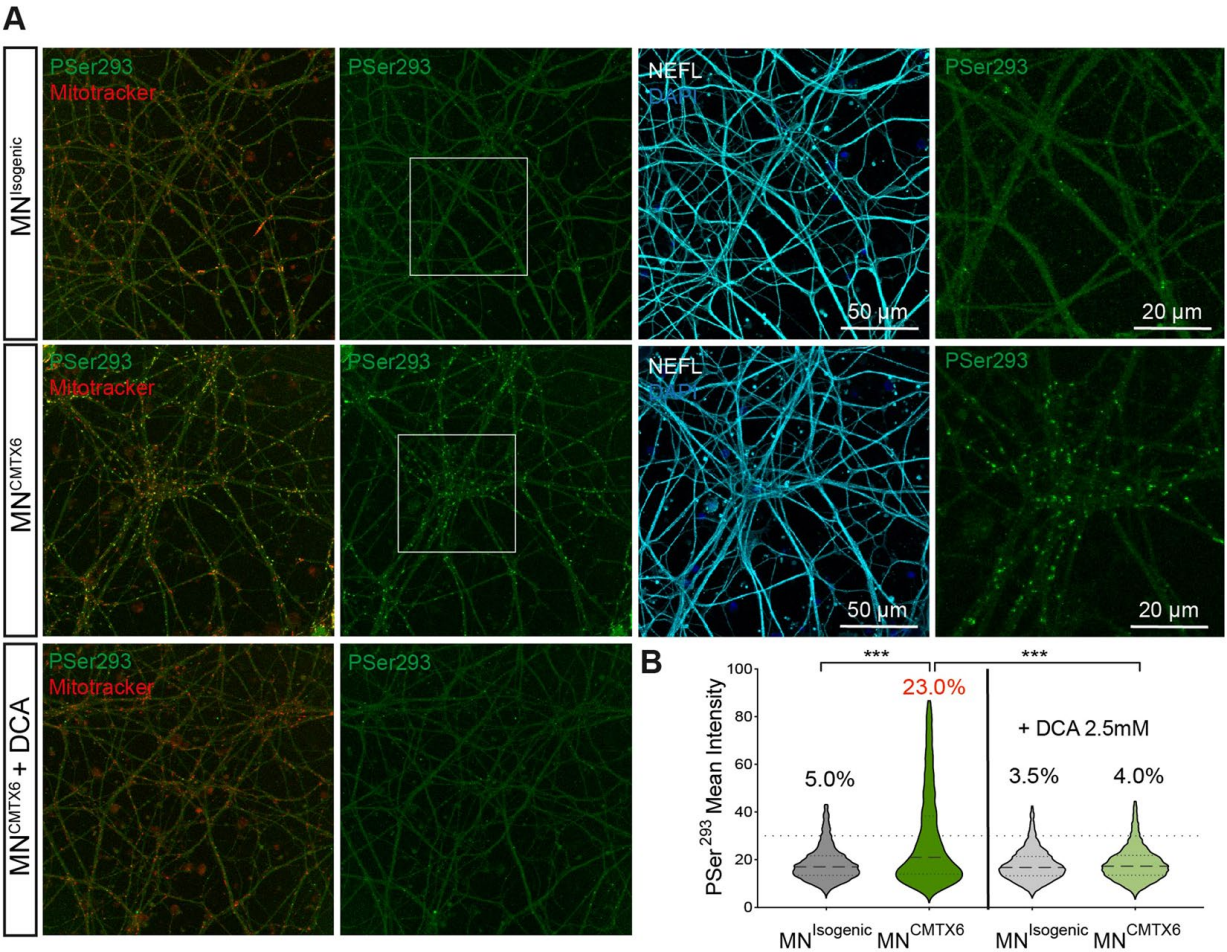
CMTX6-derived motor neurons maintain the hyperphosphorylation phenotype. (**A**) Patient MN^CMTX6^ show increased number of mitochondria with phosphorylated PDH when compared to isogenic control motor neurons (MN^Isogenic^). Treatment with DCA 5 mM for 1 h prior reverts levels of phosphorylation in the patient motor neurons. (**B**) Quantification of the mean intensity after staining the cells with the E1-PSer^293^ antibody within the region of interest (defined by staining the cells with 100 nM MitoTracker Deep Red FM). Violin plot shows the full distribution of all data points acquired (n > 5000 mitochondria). Phosphorylated mitochondria were defined as having a mean intensity above a threshold of 30 units. *p* values were obtained by ANOVA followed by Tukey’s post hoc test (***p < 0.0005).

Motor neurons were stained using the anti PSer^293^ antibody at DIV 32. Confocal microscopy revealed CMTX6 derived motor neurons (MN^CMTX6^) showed increased E1 phosphorylation when compared to the isogenic control (MN^Isogenic^). Importantly, treatment of the patient MN^CMTX6^ cells with DCA reverted phosphorylation of E1 subunit to levels found in the MN^Isogenic^ cells (Fig. 5A). To quantify this observation, all mitochondria were stained using MitoTracker Deep Red FM and the intensity of the phosphorylated Ser^293^ was measured for each stained mitochondria. We empirically established a mean threshold intensity of 30 arbitrary units to determine particles that represented phosphorylated mitochondria. In these conditions, 5.0% of all mitochondria in the MN^Isogenic^ axons were phosphorylated while 23% of the patient-derived motor neurons showed phosphorylation of the PDC at the Ser^293^ residue. Treatment with DCA showed statistically significant reduction in the number of phosphorylated mitochondria in the MN^CMTX6^ cells (Fig. 5B).

**Figure 6:**
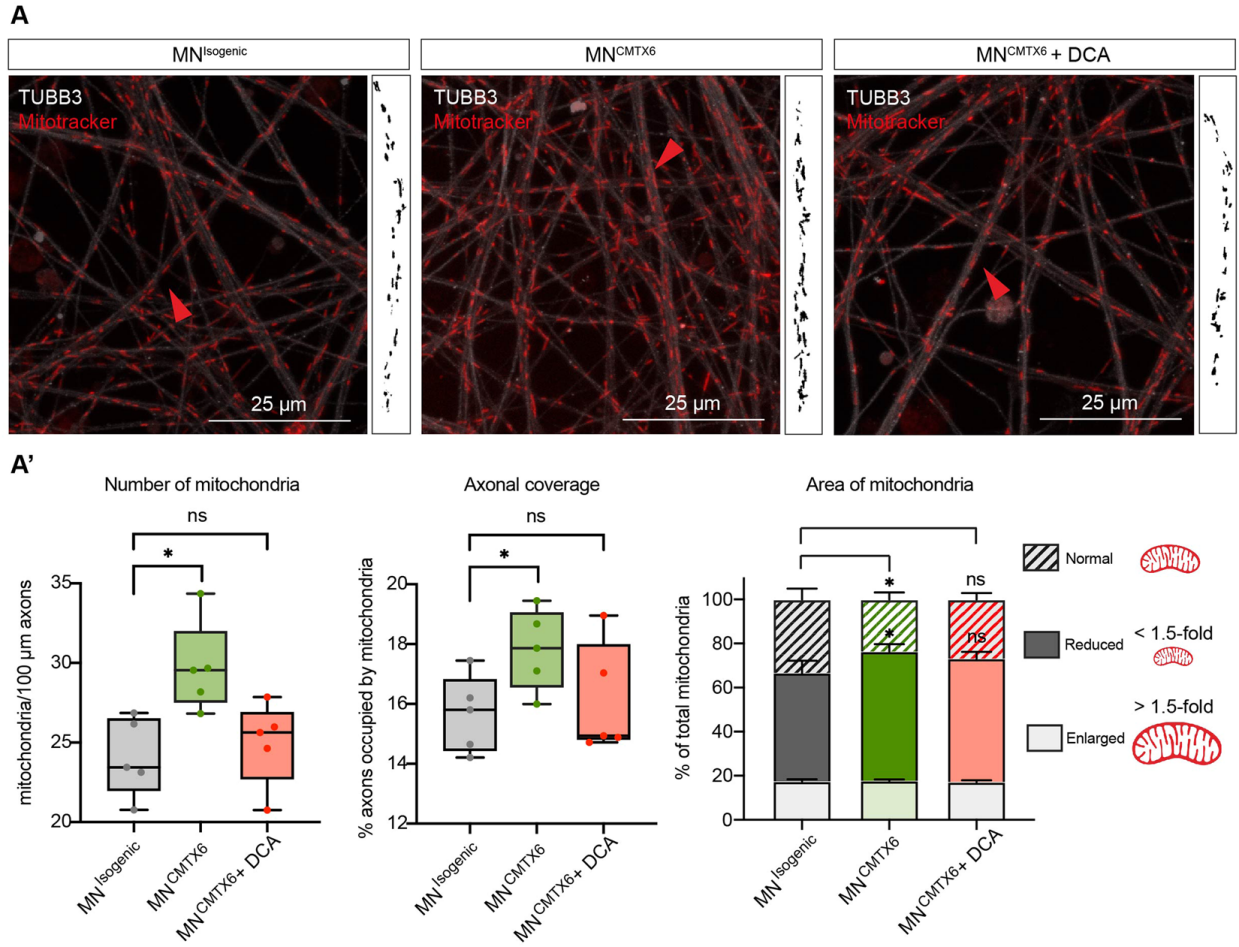
Mitochondrial features in CMTX6 patient-derived motor neurons. (**A**) Motor neurons stained with Mitotracker (red) and TUBB3 (white). Boxed area shows thresholded mitochondria within axon highlighted with the red arrow. (**A’**) Morphological features of mitochondria in MN^Isogenic^, MN^CMTX6^ and patient cells following treatment with 2.5 mM DCA for 24 h (MN^CMTX6^ + DCA). Axonal coverage was calculated by dividing the total length of all stained mitochondria by the total length of the axons. The percentage of enlarged, reduced and normal mitochondria are shown. Using the Mitotracker staining, the average area of mitochondria in MN^Isogenic^ was stablished (0.55 μm^2^). Enlarged and reduced mitochondria in the different conditions were considered those with 1.5-fold change above (> 0.83 μm^2^) or below (<0.37 μm^2^) the average mitochondria in MN^Isogenic^.

To assess distribution and morphological features of the mitochondria (Fig. 6A) the area, number and mitochondrial coverage of the axons in the MN^Isogenic^ and MN^CMTX6^ were measured. Although patient-derived motor neurons had increased numbers of stained mitochondria and a larger proportion of axons occupied with mitochondria (axonal coverage) than their isogenic counterparts, our analysis showed that a statistically significant proportion of these mitochondria were 1.5-fold smaller in the MN^CMTX6^ when compared to the MN^Isogenic^ cells. Importantly, when these morphological features were assessed in patient motor neurons following 24 h treatment with 2.5 mM DCA (MN^CMTX6^ + DCA), the number of mitochondria, axonal coverage and the percentage of reduced mitochondria were comparable to those found in the isogenic control (Fig. 6A’).

**Figure 7:**
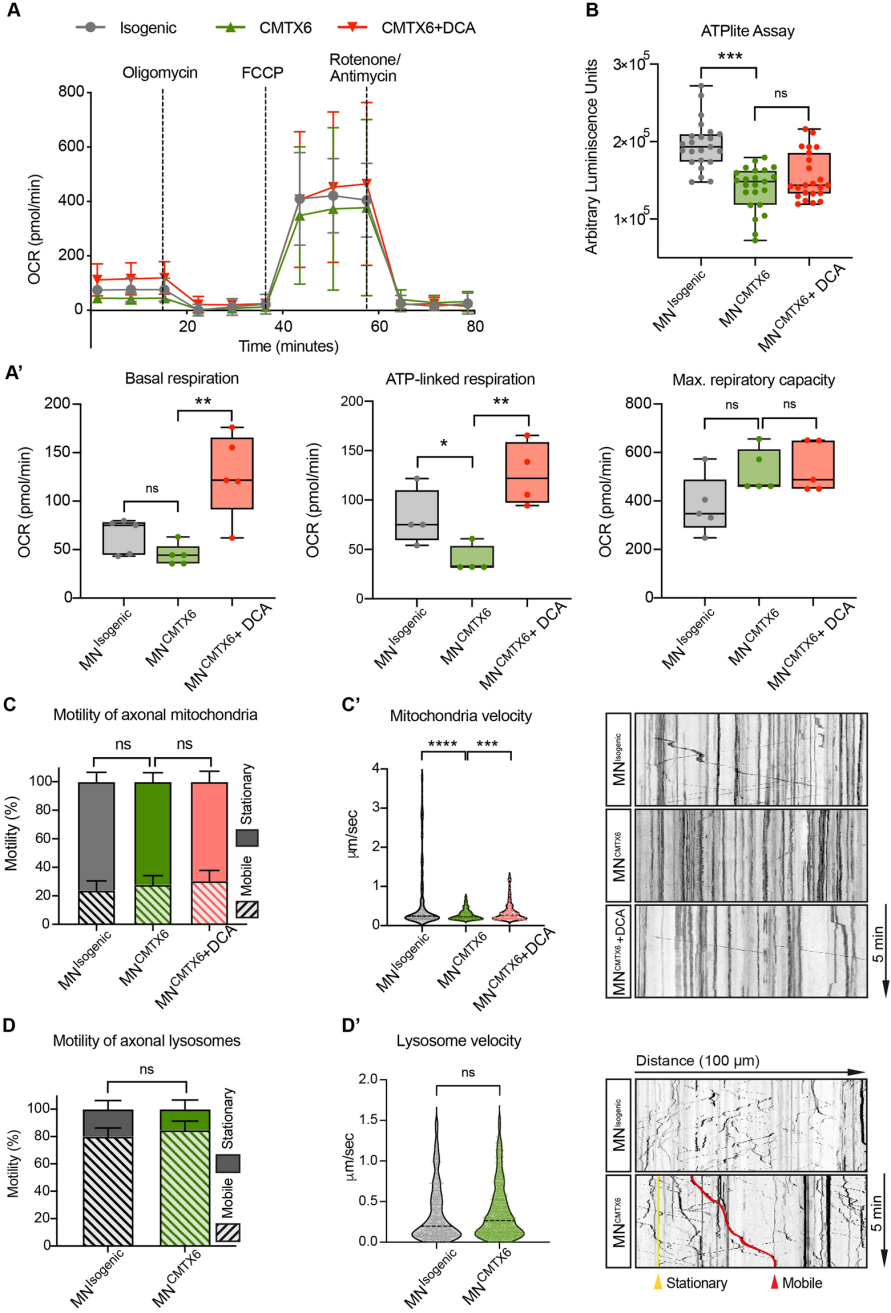
Bioenergetic profile and motility of mitochondria/lysosomes in CMTX6 patient-derived motor neurons. (**A**) Overview of the oxygen consumption rate (OCR) throughout the mitochondrial respiration test using the Seahorse XF24 Cell Mito Stress Kit. Arrows indicate the time when mitochondrial inhibitors where added to the media to assess respiratory parameters. (**A’**) OCR prior to the addition of oligomycin determines basal respiration and shows energetic demand of the cell under baseline conditions. ATP-coupled oxygen consumption was determined by inhibiting ATP synthase using oligomycin. Maximal respiration was assessed following mitochondria uncoupling by FCCP. The error bars indicate the mean ± SEM of 5 replicates from one representative experiment. (**B**) ATP production was measured using the ATPlite assay kit. Arbitrary luminescence units (ALU) are shown for each experimental group from data obtained for 3 independent experiments. (**C,D**) Motility patterns of axonal mitochondria (**C**) and lysosomes (**D**) was determined by incubating motor neurons with 200 nM Mitotracker or 100 nM Lysotracker for 45 min followed by 5-min time-lapse imaging with 2-s intervals. Kymographs for individual axons were built using the KymographBuilder plugin in *Fiji* and net displacement of individual particles over a 5 min period calculated using KymoButler Version 1.1.1. Mobile axonal particles were considered those showing net displacement greater than 10 μm. (**C’,D’**) Representative kymographs and velocity of individual mitochondria (**C’**) and lysosomes (**D’**) for each line. Data were collected from a total of 250 mobile mitochondria and 500 mobile lysosomes obtained from 10 kymographs for each line. For all experiments (**A–D**), *p* values were obtained by two-way ANOVA followed by Tukey’s post hoc test (*p < 0.05; **p < 0.01; ***p < 0.001; ****p < 0.0001).

These morphological features may be indicative of general mitochondrial dysfunction in the CMTX6-derived motor neurons. Functionally, the Seahorse Extracellular Flux Analyzer demonstrated that hyperphosphorylated mitochondria affects the metabolic profile of the CMTX6-derived motor neurons (Fig. 7A). Measurement of changes in oxygen consumption rates (OCR) following treatment of the cells with Oligomycin and FCCP revealed ATP-linked respiration is reduced in the MN^CMTX6^ when compared to the isogenic control motor neurons (Fig. 7A’). Accordingly, ATP levels in the patient motor neurons determined using the ATPlite assay, confirmed a statistically significant reduction of the ATP levels in MN^CMTX6^ when compared with MN^Isogenic^ (Fig. 7B). Treatment of the MN^CMTX6^ cells with DCA increased basal respiration and ATP-linked respiration in the patient motor neurons (Fig. 7A’), demonstrating metabolic defects in CMTX6 can be pharmacologically reverted.

Axonal transport defects have been associated with CMT and other inherited and acquired peripheral neuropathies^27^. To assess the motility of axonal mitochondria, motor neurons stained with Mitotracker were time-lapse imaged at high acquisition frequency (2-s intervals) for a total of 5 min. Analysis of 50 axons in MN^Isogenic^ (Supplementary Video. S1) revealed that mobile mitochondria (showing net displacement greater than 10 μm) represented 23.8% ± 2.1% of all mitochondria and 76.2% ± 8.7% remained stationary (Fig. 7C). No significant differences were found in the motility of axonal mitochondria in MN^CMTX6^ motor neurons (27.8% ± 2.0% mobile and 72.2% ± 7.4% stationary). Interestingly, the speed of individual mobile mitochondria (Fig. 7C’) was significantly reduced in the MN^CMTX6^ (0.27 μm/s ± 0.01) when compared with MN^Isogenic^ (0.58 μm/s ± 0.06). Detailed analysis of these particles revealed that, while 25% of mitochondria traffic faster than 0.58 μm/s in MN^Isogenic^, only 2.5% trafficked over this speed in patient-derived motor neurons (Supplementary Video. S2). Treatment of the MN^CMTX6^ cells with DCA significantly reverted this phenotype and 12.5% of all mobile mitochondria trafficked over 0.58 μm/s, showing an average speed of 0.35 μm/s ± 0.02 after treatment with the PDK inhibitor (Supplementary Video. S3).

To establish if a general axonal transport deficit is associated with the p.R158H PDK3 mutation, analysis of trafficking of axonal lysosomes in MN^Isogenic^ (Supplementary Video. S4) and MN^CMTX6^ was performed (Supplementary Fig. S5). Our data showed no difference in the proportion of mobile and stationary lysosomes between patient and control-derived motor neurons (Fig. 7D). Similarly, analysis of mobile lysosomes (Fig. 7D’) revealed no statistical differences in the speed of lysosomes in the MN^Isogenic^ (0.35 μm/s ± 0.02) when compared with MN^CMTX6^ (0.37 μm/s ± 0.01). This data suggests the altered distribution of mitochondria and the reduced speed of mobile mitochondria seen in MN^CMTX6^ is a consequence of the mitochondrial dysfunction and it is not a result of a general axonal transport deficit in CMTX6 motor neurons.

## Discussion

**Figure 8:**
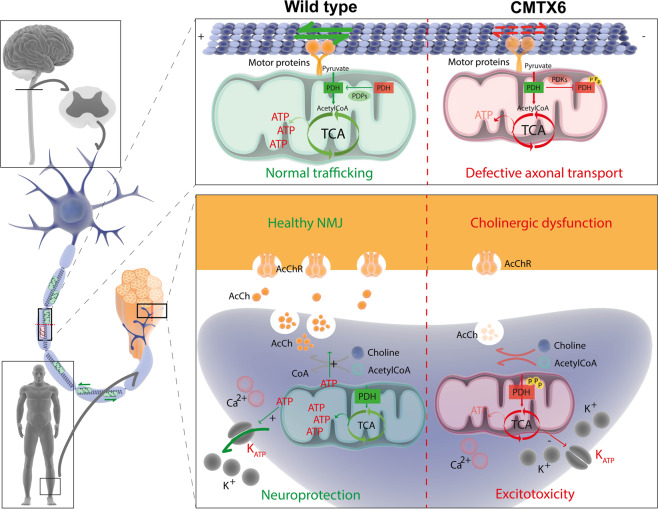
Motor neuron integrity and synaptic activity relies on maintaining energy production and mitochondrial function in distal axons. Length-dependent axonal degeneration is a pathological hallmark of CMT neuropathies. Long axons of the peripheral nervous system are likely to be more susceptible to deficient axonal transport of mitochondria and other cargoes to the neuromuscular junction. This is an energy-dependent task that relies on motor proteins and adaptors that assist in the anterograde transport towards the terminal end (kinesins and kinesin-1) and back to the cell body (retrograde transport, mediated by the dynactin/dynein complex). At the synapse, ATP production, release and activation of the P2Y/K_ATP_ cascade elicits the opening of K_ATP_ channels. This has a neuroprotective effect through hyperpolarization of neurons and a decrease in neuronal excitability^32^. At the neuromuscular junction (NMJ), synaptic activity is sustained by activation of acetyl choline receptors (AcChR). Synthesis of acetyl choline (AcCh) relies in the sufficient availability of cytosolic ATP and the reduced release of AcCh has been observed in mouse models of CMT^33^. Reduced availability of ATP caused by the p.R158H mutation may impact any of these cellular pathways and could therefore be the pathomechanism underlying degeneration of distal axons in CMTX6. Ilustrations for drawing this figure were obtained from the “Library of Science and Medical Ilustrations” (https://www.somersault1824.com/resources).

Maintenance of bioenergetic homeostasis is crucial for structural and functional integrity of distal axons. Disruption of energy‐dependent axonal transport as a mechanism for length-dependent distal axonal degeneration was first proposed nearly 40 years ago^28^, before any of the genes responsible for inherited peripheral neuropathies had been identified. There is now strong genetic evidence for this assertion. Due to their unique morphology, long peripheral nerves require effective maintenance of ATP levels to support energy consuming activities, including axonal transport and the generation of ion gradients at the end terminal (Fig. 8). The discovery of *PDK3* as the CMTX6 causative gene has added to the growing list of CMT genes related to mitochondrial biology^5^, providing further genetic evidence that mitochondrial pathway deficits may be a common theme of pathogenic mechanism in CMT neuropathies.

Our previous investigation using patient fibroblasts from the family initially describing the CMTX6 mutation, showed that increased kinase activity of the mutant PDK3^3^ led to hyperphosphorylation of the E1 subunit of the PDC and consequently attenuation of the complex activity^6^. The report of a second unrelated family with the same genetic mutation, demonstrated the p.R158H as a mutational “hotspot” in the *PDK3* gene^4^. Experiments presented in this study showing E1 hyperphosphorylation in the two unrelated patient-derived fibroblast lines has provided compelling evidence confirming this as a CMTX6 phenotypic signature (Fig. 1). This phenotype is maintained in patient-derived iPSC (Fig. 3A) and yet, clinical examination of CMTX6 patients demonstrates only distal axons are affected by increased PDK3 activity^3,4^, thus reinforcing the notion that maintaining bioenergetic homeostasis is essential to preserve the integrity of the long peripheral axons.

Despite our increased understanding of the causative genes contributing to CMT and recognising the importance of bioenergetic pathway defects that contribute to the pathology of the disease, to date no effective treatment is available for patients. This is in part due to the absence of relevant disease models^29^ and well-defined cellular phenotypes that can be interrogated as a proxy for drug efficacy^30^. Data presented in this study demonstrates our approach provides the necessary platform to overcome these limitations. Our experiments using the pan PDK inhibitor DCA demonstrate the E1 hyperphosphorylation (Fig. 5A) and the associated metabolic and mitochondrial abnormalities (Figs. 6 and 7) seen in the CMTX6 patient-derived motor neurons can be pharmacologically reverted to the wild type phenotype. Although the use of DCA in a clinical setting is not advisable since worsening of peripheral neuropathy and tumor growth after high doses have been reported^31^, our results provide a proof of concept for high content screening of molecules for therapeutic application.

Our data shows the *PDK3* mutation causes changes in the morphological features and distribution of the mitochondria in the patient-derived motor neurons, in line with our previous investigations using CMTX6 fibroblasts^6^. Our experiments showing the increased proportion of smaller mitochondria in the CMTX6 motor neurons (Fig. 6) suggested mitochondria fragmentation might be an underlying consequence of the p.R158H PDK3 mutation. Functionally, these observations correlate with mitochondrial dysfunction in the patient motor neurons, as demonstrated by the reduced capacity of the mutant cells to sustain mitochondria respiration (Fig. 7A,B), thereby suggesting energy deficits may be a primary cause of pathology in CMTX6. Unlike mitochondrial axonal trafficking (Fig. 7C), lysosomal movement remains unaltered in the patient motor neurons (Fig. 7D) suggesting the p.R158H PDK3 mutation does not cause general axonal transport defects. Our data suggests the inefficient supply of ATP at the nerve terminal leading to synaptic deficits may be the underlying cause of axonal degeneration in CMTX6 (Fig. 8). Further studies, including neurophysiologic analysis to assess increased excitability in the patient motor neurons as a result of dysfunctional ATP-dependent ion channels will provide an ideal platform to investigate this hypothesis.

Despite our data demonstrating metabolic dysfunction in the CMTX6 derived motor neurons, no signs of axonal damage were found for the patient axons at the time-points interrogated for this study (DIV 32, DIV 37 and DIV 42). Axonal degeneration is the main pathological characteristic seen in CMT patients and modelling this feature using iPSC-derived neurons remains the biggest challenge in developing cellular models of CMT. A major advance towards this goal is the recent report of human tridimensional neuronal cultures (spinal spheroids) that allows spatial separation of individual extending axons in a centrifugal fashion^15^. Using this approach, Maciel el al. achieved continuous growth of isolated neurites that reached 1 cm in length after being 50 days in culture. Future research using this platform may reveal differential length-dependent axonal degeneration of CMTX6-derived spinal spheroids.

In conclusion, we have stablished a CMTX6 iPSC motor neuron cell line. Patient motor neurons display hyperphosphorylation of the E1 subunit of PDC when compared with isogenic control cells and display mitochondrial and bioenergetic abnormalities. The fact that the cellular phenotype described in the patient motor neurons can be pharmacologically reverted will facilitate identifying therapeutic alternatives for CMTX6 patients. Based on the evidence that impaired metabolic capacity is a cellular hallmark of diseased axons in inherited peripheral neuropathies, our CMTX6 motor neuron model may also be utilised for screening approaches in other forms of CMT.

## Supplementary information

Supplementary information.

Supplementary Video S1.

Supplementary Video S2.

Supplementary Video S3.

Supplementary Video S4.

Supplementary Video S5.

## Data Availability

The datasets generated in the current study are available from the corresponding author on request.
