# Supplementary material for: Energy metabolism and mitochondrial defects in X-linked Charcot-Marie-Tooth (CMTX6) iPSC-derived motor neurons with the p.R158H PDK3 mutation
Source: Sci Rep. 2020 Jun 5;10:9262. doi: 10.1038/s41598-020-66266-5 (PMC7275085; doi:10.1038/s41598-020-66266-5)

SUPPLEMENTARY INFORMATION

Energy metabolism and mitochondrial defects in X-linked Charcot-Marie-Tooth (CMTX6) iPSC-derived motor neurons with the p.R158H PDK3 mutation.

Perez-Siles G [1, 2, *], Cutrupi A [1, 2], Ellis M [1], Screnci R [4], Mao D [5], Uesugi M [5], Eppie M. Yiu [6, 7, 8], Monique M. Ryan [6, 7, 8], Choi BO [9], Nicholson G [1, 3], Kennerson M L [1, 2, 3, *].

[1] Northcott Neuroscience Laboratory, ANZAC Research Institute, Sydney, Australia;

[2] Sydney Medical School, University of Sydney, Sydney, Australia;

[3] Molecular Medicine Laboratory, Concord Repatriation General Hospital, Sydney, Australia;

[4] School of Life Sciences, University of Technology Sydney, Sydney, NSW, Australia;

[5] Institute for Integrated Cell-Material Sciences and Institute for Chemical Research, Kyoto University, Japan.

[6] Department of Neurology, Royal Children's Hospital, Flemington Road, Parkville, VIC, Australia.

[7] Neuroscience Research, Murdoch Children's Research Institute, Melbourne, VIC, Australia.

[8] Department of Paediatrics, The University of Melbourne, VIC, Australia.

[9] Department of Neurology, Samsung Medical Center, Sungkyunkwan University School of Medicine, Seoul, Korea.

* Correspondence: marina.kennerson@sydney.edu.au
gonzalo.perez-siles@sydney.edu.au

Supplementary Movie 1. $MN^{Isogenic}$ at DIV32 were stained with 200 nM MitoTracker Deep Red for 45min and, following replacement with fresh medium, trafficking organelle imaged at 63X magnification with 2-s intervals for 5-min.

Supplementary Movie 2. MN^{CMTX6} at DIV32 were stained with 200 nM MitoTracker Deep Red for 45min and, following replacement with fresh medium, trafficking organelle imaged at 63X magnification with 2-s intervals for 5-min.

Supplementary Movie 3. MN^{CMTX6} at DIV31 were incubated with 2,5mM DCA for 24 hours. At DIV 32 motor neurons were stained with 200 nM MitoTracker Deep Red for 45min and, following replacement with fresh medium, trafficking organelle imaged at 63X magnification with 2-s intervals for 5-min.

Supplementary Movie 4. $MN^{Isogenic}$ at DIV32 were stained with 100 nM LysoTracker Deep Red for 45min and, following replacement with fresh medium, trafficking organelle imaged at 63X magnification with 2-s intervals for 5-min.

Supplementary Movie 5. MN^{CMTX6} at DIV32 were stained with 100 nM LysoTracker Deep Red for 45min and, following replacement with fresh medium, trafficking organelle imaged at 63X magnification with 2-s intervals for 5-min.

Original blots displayed in Fig 2E. Western blot demonstrates expression of pluripotency markers in the iPSCs (lines 1 and 2) and not in the CMTX6 patient fibroblasts (line 3). Membranes incubated with Nanog (membrane 1), Sox2 (membrane 2) and Oct-4A (membrane 3). All membranes were blotted against β-actin as a loading control for this experiment.

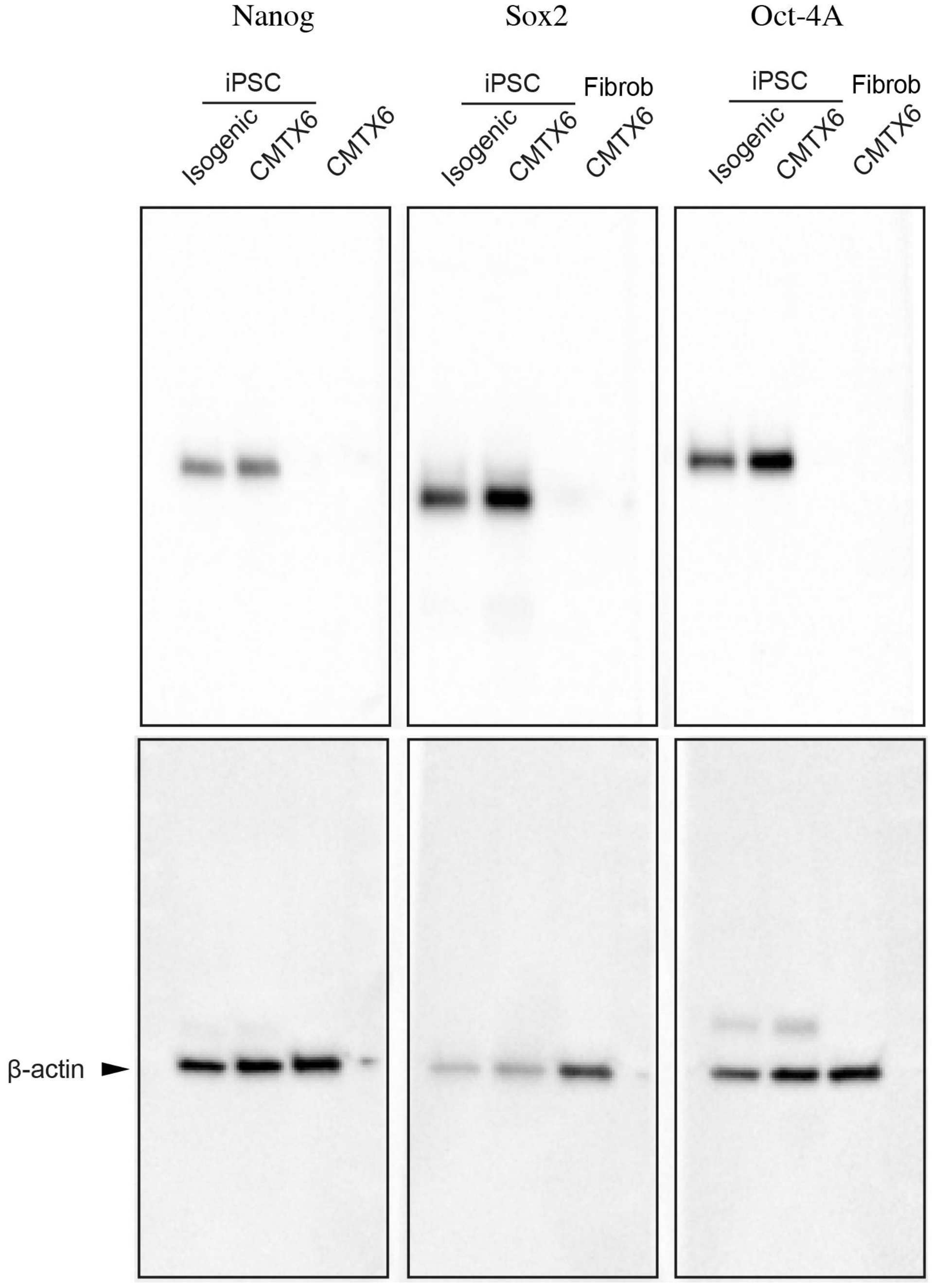

Supplement: Supplementary file 1 — Supplementary information. [file 41598_2020_66266_MOESM1_ESM.pdf]
